# Molecular Model of Plasma PAF Acetylhydrolase-Lipoprotein Association: Insights from the Structure

**DOI:** 10.3390/ph3030541

**Published:** 2010-03-08

**Authors:** Prabhavathi Srinivasan, Brian J. Bahnson

**Affiliations:** Department of Chemistry & Biochemistry, University of Delaware, Newark, DE, 19716, USA

**Keywords:** PAF-AH, Lp-PLA_2_, group VIIA PLA_2_, lipoprotein, i-face

## Abstract

Plasma platelet-activating factor acetylhydrolase (PAF-AH), also called lipoprotein-associated phospholipase A_2_ (Lp-PLA_2_), is a group VIIA PLA_2_ enzyme that catalyzes the hydrolysis of PAF and certain oxidized phospholipids. Although the role of PAF-AH as a pro- or anti-atherosclerotic enzyme is highly debated, several studies have shown it to be an independent marker of cardiovascular diseases. In humans the majority of plasma PAF-AH is bound to LDL and a smaller portion to HDL; the majority of the enzyme being associated with small dense LDL and VHDL-1 subclasses. Several studies suggest that the anti- or pro-atherosclerotic tendency of PAF-AH might be dependent on the type of lipoprotein it is associated with. Amino acid residues in PAF-AH necessary for binding to LDL and HDL have been identified. However our understanding of the interaction of PAF-AH with LDL and HDL is still incomplete. In this review we present an overview of what is already known about the interaction of PAF-AH with lipoprotein particles, and we pose questions that are yet to be answered. The recently solved crystal structure of PAF-AH, along with functional work done by others is used as a guide to develop a model of interaction of PAF-AH with lipoprotein particles.

## 1. Introduction

**Figure 1:**
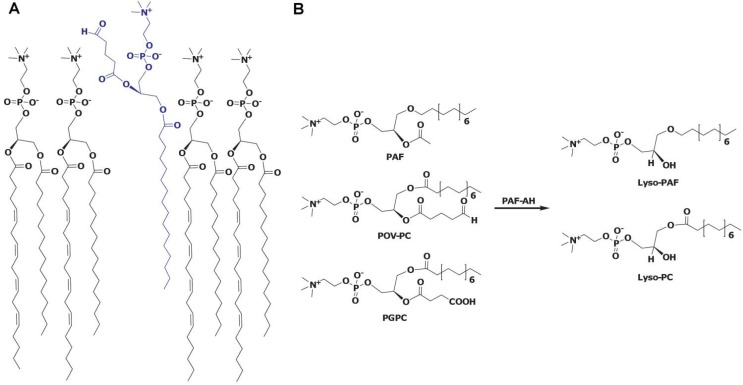
PAF-AH substrates. Panel A depicts a model of the phospholipid monolayer of lipoproteins with an oxidatively fragmented phospholipid (shown in blue). The *sn-2* chain of the fragmented phospholipid could be flipped upward or away from the hydrophobic portion of the interface and thus become accessible to the PAF-AH active site. Panel B shows examples of different types of PAF-AH phospholipid substrates (PAF; POV-PC: 1-(palmitoyl)-2-(5-oxovaleroyl)-phosphatidylcholine; PGPC: 1-palmitoyl-2-glutaroyl phosphatidylcholine) that are hydrolyzed to give the product free fatty acid and either lyso-phosphatidylcholine (lyso-PC) or lyso-PAF.

Human platelet-activating factor acetylhydrolase (PAF-AH) is a Ca^2+^ independent phospholipase A_2_ (PLA_2_) that was identified in human plasma as the enzyme responsible for the hydrolysis and thus inactivation of platelet-activating factor (PAF, 1-O-hexa/octadecyl-2-acetyl-*sn*-glycero-3-phospho-choline) [1,2]. PAF is a potent pro-inflammatory phospholipid signaling molecule [3]. Specifically, PAF-AH catalyses the hydrolysis of the acetyl group at the *sn-2* position of the glycerol backbone of PAF converting it to lyso-PAF (Figure 1). Due to its PLA_2_ type catalytic activity the enzyme is also referred to as group VIIA PLA_2_ [4]. Both intracellular as well as secreted forms of PAF-AH have been identified [5,6]. One of the intracellular forms, homologous to the secreted PAF-AH, is referred to as PAF-AH II or group VIIB PLA_2_ [4]; this article deals with only the secreted plasma PAF-AH.

The enzyme has a classic lipase α/β-hydrolase fold with a Ser, Asp, His catalytic triad, as confirmed by the recently solved crystal structure of the enzyme [7]. In addition to PAF, the enzyme catalyzes the hydrolysis of a wide variety of substrates [8], including oxidatively fragmented phospholipids produced as a result of LDL oxidation (Figure 1). 

Typically, only phospholipids with a short acyl chain, up to 5 carbons long at the *sn*-2 position are hydrolyzed. The enzyme can hydrolyze phospholipids with longer acyl chains, if they have oxidized functionality at the ω-position [9]. Due to this unique substrate specificity the enzyme can circulate freely in the plasma in the active form without hydrolyzing cellular phospholipids. In the plasma, the enzyme is bound to lipoprotein particles [10] and is therefore also known as lipoprotein associated phospholipase A_2 _(Lp-PLA_2_).

## 2. Physiological Role of Plasma PAF-AH

A large number of studies have been published over the years since plasma PAF-AH was first discovered linking an increase in plasma PAF-AH concentration or activity to an increased risk of various cardiovascular diseases [11,12]. This has prompted many researchers to suggest the use of plasma PAF-AH as a biomarker for assessing risk of future coronary heart diseases (CHD). The use of plasma PAF-AH as a biomarker for predicting CHD has been the subject of a number of recent reviews [12,13,14,15] and will not be discussed in detail in this article. Although many studies have found a correlation between plasma PAF-AH and CHD, it is not clear if plasma PAF-AH is the causative agent or simply a marker of inflammation. The biological function of plasma PAF-AH in the development of CHD is controversial, with both anti- and pro-inflammatory roles attributed to it. Plasma PAF-AH is thought to be anti-inflammatory due to its ability to hydrolyze bio-active, pro-inflammatory oxidatively fragmented phospholipids produced during the oxidation of LDL. The enzyme is therefore thought to play a protective role in CHD, preventing the accumulation of these bio-active molecules on LDL. The observed correlation between plasma PAF-AH concentration or activity and CHD is thought to be a physiological response to increased vascular inflammation. On the other hand, hydrolysis of the bio-active molecules by plasma PAF-AH results in the formation of lysophosphatidylcholine and oxidized nonesterified fatty acids, molecules that have been shown to be highly effective proatherogenic inflammatory mediators [16,17,18,19]. It is believed that plasma PAF-AH mediated hydrolysis causes the accumulation of these molecules and aids in atherosclerotic plaque development. Epidemiological, genetic and animal model studies give varying indications about the role played by plasma PAF-AH in CHD. There are a number of studies supporting the role of plasma PAF-AH as both a pro- and anti-inflammatory enzyme [20,21]. A consensus view of prior published work is that only the LDL-associated plasma PAF-AH is pro-atherosclerotic, while the HDL-associated plasma PAF-AH is anti-atherosclerotic. 

**Figure 2:**
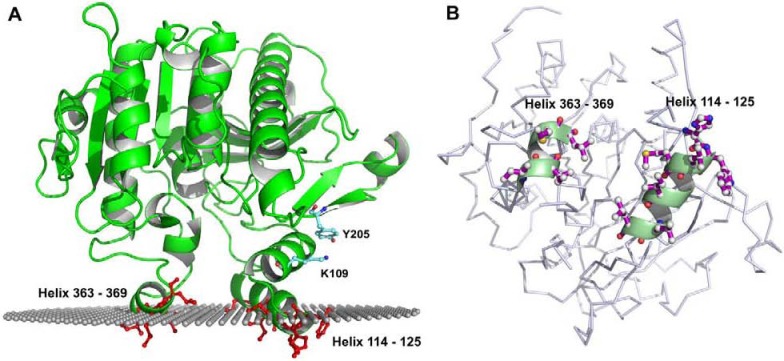
Orientation of PAF-AH on a membrane surface as predicted by OPM. Panel A shows the orientation of plasma PAF-AH on a membrane surface. The i-face residues are shown in red. Also shown is K109 and Y205 which display a cation-pi interaction. A list of all the i-face amino acids is provided in Table 1. The OPM derived plane represents a region of intermediate hydrophobicity; the hydrophilic head groups extend ~10Å above the plane of spheres. Panel B shows the i-face residues after a ~90˚ rotation of the view from panel A.

The focus of this review is on the interaction of plasma PAF-AH with lipoprotein particles. The current understanding is that distinct regions of plasma PAF-AH interact with LDL and HDL particles. However, it is not clear how the two regions mediate the interaction with LDL and HDL particles. The crystal structure of plasma PAF-AH has allowed us to further develop the current understanding of its interaction with lipoprotein particles. We have developed a model of PAF-AH bound to an aqueous-lipid interface using the methods of the orientation of proteins in membranes (OPM) [22,23]. The result of the OPM method (prediction performed by A. Lomize) is presented in Figure 2. The plane represents the interface between polar and nonpolar components, and the polar head groups of the phospholipids are believed to extend ∼10 Å above the plane shown. Using the orientation of PAF-AH predicted by OPM and the surface electrostatic potential view of PAF-AH, we can now propose a model of the interaction of plasma PAF-AH with LDL and HDL particles. Both LDL and HDL are complexes of lipids and proteins, consisting of a core of triglycerides and cholesteryl esters covered by a surface monolayer composed of phospholipids, unesterified cholesterol and apolipoproteins. 

**Table 1:** Plasma PAF-AH amino acids predicted to interact with lipoproteins LDL and HDL.

i-face amino acids	Charged amino acids	
	Basic Patch	Acidic Patch
**Helix 114–125:** H114*, W115*, L116*, M117*, I120, L123, L124 **Helix 363–369:** I364, I365, M368*, L369*	K55, R58, K363, K101, R122 H367*, K370*	D374, D376, D382, D401, D403, D406, D412, D413, E414

This Table lists amino acids predicted to be important for the association of plasma PAF-AH with LDL and HDL, based on the structure. Residues marked with * have previously been identified as part of either a HDL binding [41] or LDL binding [33] region.

We believe that distinct amino acids of plasma PAF-AH are involved in its interaction with both the lipid and protein components of lipoproteins. Two plasma PAF-AH α-helices rich in hydrophobic amino acids likely interact with the phospholipid monolayer of both LDL and HDL particles. A cluster of negatively charged amino acids present on plasma PAF-AH is proposed to mediate an interaction with the protein component (apoB100) of LDL particles. Furthermore, the PAF-AH structure reveals two regions rich in negatively charged residues, which may mediate its interaction with either the protein or lipid component of LDL and HDL. The role played by each of these regions and the type of interaction mediated by them in the association of plasma PAF-AH with LDL and HDL particles will be discussed in detail.

## 3. Plasma PAF-AH Association with Lipoproteins

In the plasma, PAF-AH is bound to lipoprotein particles, a majority to LDL and a smaller percentage to HDL. The actual percentage bound to each of the two particles seems to vary depending on the method used for isolating the lipoprotein particle. When ultracentrifugation was used to isolate lipoprotein particles, about 70% of the plasma PAF-AH activity is associated with the LDL fraction. The remainder was in the HDL fraction and in the lipoprotein depleted (d > 1.21g/ml) fraction [10,24,25]. When size-exclusion chromatography was used to separate lipoprotein particles, 85% PAF-AH was found in the LDL fraction and the remaining 15% in the HDL fraction [24]. This observation of variable distribution of plasma PAF-AH led to the suggestion that two types of plasma PAF-AH might exist, one population that binds tightly to LDL and another loosely bound population that dissociates during ultracentrifugation and moves to higher density fractions.

### 3.1. Plasma PAF-AH binding to LDL

Within these two classes of lipoproteins, the enzyme has been shown to favor certain subclasses. Plasma PAF-AH preferentially binds to small dense LDL particles and very-high-density lipoprotein–1 subfraction [26,27]. Also it was demonstrated that PAF-AH is mainly associated with LDL(-) [28], a LDL subfraction with increased electronegative charge. LDL(-) is elevated in individuals with increased cardiovascular risk and has been shown to have pro-inflammatory and cytotoxic effects on endothelial cells (reviewed in [29]). Furthermore, in individuals with elevated Lp(a) levels, plasma PAF-AH has been shown to have a higher affinity for Lp(a) than to LDL [30]. Lp(a) is a lipoprotein differing from LDL in having an additional glycoprotein apo(a), linked by a disulphide bond to apoB100. Lp(a) is proposed to be the carrier of pro-inflammatory oxidized phospholipids and an elevated Lp(a) level has been shown to be associated with an increased risk of cardiovascular diseases (reviewed in [31,32]). The reason for increased affinity of plasma PAF-AH for these apparently atherogenic subfractions has not been determined. ApoB100, the only apolipoprotein present in LDL, has been shown to mediate the interaction of plasma PAF-AH with LDL [33]. It is possible that the altered conformation of apoB100 in the LDL subfractions discussed above promotes binding of plasma PAF-AH. Indeed, the enhanced association of plasma PAF-AH with Lp(a) was shown to be due to the increased affinity to Lp(a)-apoB100 [30]. The increased affinity of plasma PAF-AH for small dense LDL as well as LDL(-) has also been proposed to be mediated by an increased affinity to apoB100, as a result of its altered conformation [26,34]. The physiological significance of enrichment of plasma PAF-AH in these subfractions is unclear. The preferential association of plasma PAF-AH with these apparently pro-atherogenic subfractions is considered to be further support for the pro-atherogenic character of plasma PAF-AH. However, studies demonstrating a clear role of plasma PAF-AH in atherogenicity are lacking and arguments are presented for both a pro- and an anti-inflammatory role of the enzyme in these sub-fractions.

### 3.2. Plasma PAF-AH binding to HDL

Contrary to the controversial role of LDL-bound PAF-AH, HDL-bound PAF-AH is consistently reported to be anti-atherosclerotic. PAF-AH was shown to contribute significantly to the protective effect of HDL in preventing oxidation of LDL [35]. Catalytic activities of three different enzymes on HDL: plasma PAF-AH, paraoxonase 1 (PON1) and LCAT, are believed to contribute towards its protective role against oxidation of LDL and inhibition of cell stimulation induced by oxidized LDL (reviewed in [36]). However it was later demonstrated that the anti-inflammatory role attributed to PON1 was actually due to the co-purification of a small amount of plasma PAF-AH [37]. In addition, adenoviral mediated human plasma PAF-AH gene transfer in apoE^-/-^ mice has been shown to have anti-atherosclerotic effects [38,39]; since the majority of plasma PAF-AH in mouse is bound to HDL [40,41] this was considered as further proof of an anti-atherosclerotic role of HDL-bound plasma PAF-AH. However, Noto *et al.* later found that the adenovirus mediated transfer of human plasma PAF-AH gene to apoE-/- mice resulted in PAF-AH that bound to all the lipoprotein particles and provided protection against oxidative stress [42].

The ratio of LDL- to HDL-bound plasma PAF-AH has been demonstrated to increase in different diseases. Patients with paroxysmal atrial ﬁbrillation had an increased amount of LDL bound plasma PAF-AH and a lower amount bound to HDL compared to the control group [43]. This result can be thought of as an increased ratio of LDL to HDL bound PAF-AH enzyme. In individuals with hypercholesterolemia the enzyme activity associated with small dense LDL increased, whereas the HDL associated activity remained unchanged [44,45]. These observations led to the proposal that the ratio of plasma PAF-AH bound to HDL versus that bound to the total lipoproteins could be used as a marker of the severity of inflammation in different diseases. However in a different study, the HDL-bound concentration of PAF-AH was significantly higher; whereas the PAF-AH concentration not associated with HDL was lower in hyperlipidemic and diabetic subjects than in controls [46]. Taken together these studies indicate that the role of plasma PAF-AH on HDL is not clearly established yet and further studies are required before a conclusion can be reached. The same enzyme is present on LDL and HDL, and considering that the enzyme activity is not affected by binding to either lipoprotein particles [8], the reason for the HDL bound enzyme to be anti-atherosclerotic and the LDL-bound enzyme to be pro-atherosclerotic is not clear. 

The factors that influence the distribution of PAF-AH between the two lipoproteins are not clearly established. However, there are important studies that provide clues to the enzyme favoring one particle over another. Tselepis *et al.* demonstrated that the N-linked glycosylation in PAF-AH hinders its binding to HDL [25]; the removal of glycosylation enhanced the enzyme’s association with HDL. Thus, the degree of glycosylation appears to be an important factor that influences the distribution of enzyme among different lipoprotein fractions. In addition, as mentioned above, it has been shown that the apoB100 present in LDL, but not in HDL, mediates the binding of PAF-AH to LDL [33]. Additional features of PAF-AH that might be influencing its distribution became apparent from its structure, as discussed below.

## 4. Current Knowledge of the Determinants of PAF-AH Binding to Lipoproteins

Distinct regions of plasma PAF-AH have been proposed to be involved in binding to LDL and HDL [33,41]. Specific N-terminal amino acid residues of PAF-AH were identified to be essential for its interaction with LDL; these include: Y205, W115, L116 and to a lesser extent, M117. Changing all these hydrophobic residues to alanine reduced binding to LDL drastically [33]. Mouse plasma PAF-AH does not have Trp and Leu at positions corresponding to W115 and L116, and does not bind to LDL. Replacing the residues corresponding to 115 and 116 in mouse to Trp and Leu, rendered the enzyme capable of binding to LDL. Another mutation that affected binding was H114A. However, it was shown that H114 is not directly involved in the binding but is present close to the residues important for binding. The pH dependent binding to LDL that plasma PAF-AH exhibits [10], is due to the charged state of the H114 present in the binding surface.

More recently, C-terminal amino acid residues HMLK (367–370) were identified as being important for binding to HDL particles. Among the four residues a more prominent role for M368 and L369 was suggested while H367 and K370 were suggested to play a moderate role in binding [41]. As has been mentioned earlier, the carboxy terminal region of apoB100 present on LDL is also believed to be involved in the binding of plasma PAF-AH with LDL [33]. However, the region in PAF-AH that interacts with the protein component of LDL has not yet been identified. Each of these regions involved in PAF-AH-lipoprotein interaction will be discussed below in further detail in light of the structure of PAF-AH.

## 5. Molecular Model of Lipoprotein-PAF-AH Association: Hints from the Structure

### 5.1. Role of Hydrophobic and Aromatic Residues

The recently solved crystal structure of plasma PAF-AH [7] together with the OPM methods of Lomize *et al.* [22,23] were used to predict its association with a membrane surface. The OPM method determines the orientation of membrane proteins in membranes by minimizing their transfer energy from water to a lipid bilayer. The method can distinguish between membrane proteins and soluble proteins based on their transfer energy and membrane penetration depths. Figure 2, shows the orientation of plasma PAF-AH as predicted by the OPM method. This prediction was used as a basis to develop the model of PAF-AH associating with the phospholipid monolayer of LDL and HDL lipoproteins. The surface of a peripheral membrane protein that interacts with the membrane is referred to as the interface binding surface (i-face). The i-face of plasma PAF-AH consists of two short α-helices rich in hydrophobic residues that presumably insert into the interfacial region of the membrane. As discussed above many of these i-face residues have been shown by site-directed mutagenesis to be important for binding to lipoproteins. The amino acids important for binding to LDL are part of the N-terminal α –helix, while the amino acids involved in binding to HDL are part of the C-terminal α –helix. The OPM prediction identified additional residues in the i-face α –helices that are likely to be involved in PAF-AH-LDL association (Table 1).

**Figure 3:**
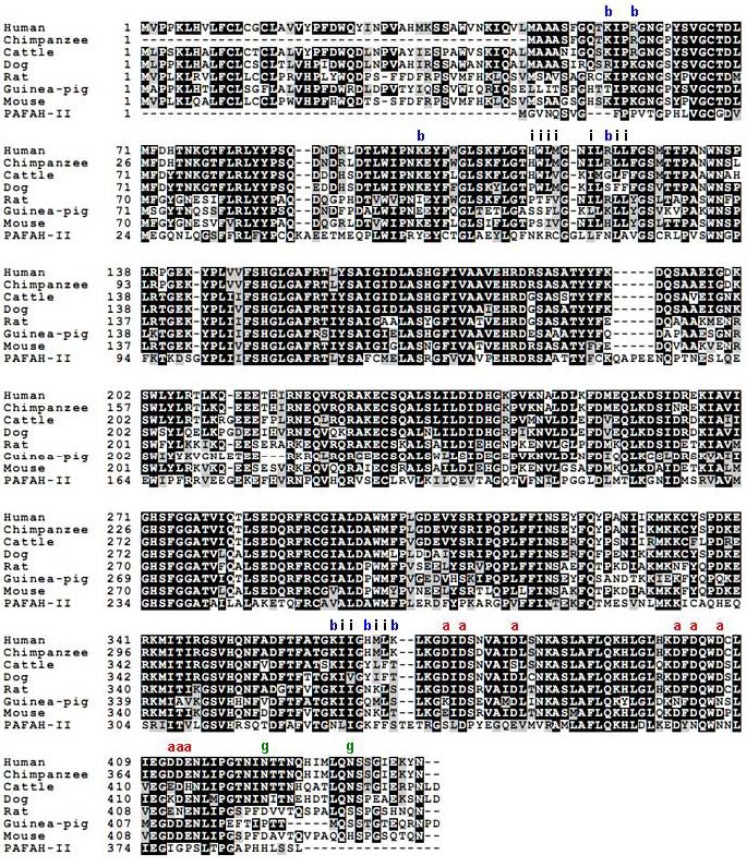
Alignment of PAF-AH amino acid sequences from different mammals. Amino acid sequence of human PAF-AH II is also included for comparison. Amino acids comprising the i-face, acidic patch and basic patch regions of plasma PAF-AH are labeled with the letters – i, a and b respectively. Glycosylation sites are labeled with the letter g.

As shown in Figure 3, a comparison of plasma PAF-AH sequences from different species showed that these additional i-face residues are well conserved.

Although the model of PAF-AH bound to a membrane surface predicted by OPM is in agreement with the functional studies carried out to date, there are two important inconsistencies. As mentioned above, distinct regions of PAF-AH are believed to interact with LDL and HDL particles. However, from the structure of plasma PAF-AH, no features are apparent that support the idea that distinct regions of PAF-AH separately interact with LDL and HDL. We propose that the two i-face α-helices together are involved in binding to both LDL and HDL lipoproteins. In this regard, it should be noted that in both the studies that separately identified the LDL and HDL binding regions in PAF-AH, the binding experiments were done exclusively with one lipoprotein particle only. Unless the effect of mutating the relevant residues on both the lipoprotein particles is studied in parallel, it cannot be ruled out that the same regions of the enzyme interact with both the lipoprotein particles.

### 5.2. Role of Y205

The other disagreement with the current understanding of plasma PAF-AH lipoprotein interaction is regarding the role of Y205. As mentioned above, Y205A mutation has been shown to abolish the binding of PAF-AH to LDL. However in the OPM model, the Y205 residue is predicted to be well above the plane of the membrane, and therefore too far away to be directly involved in binding (Figure 2, panel B). One possible explanation for the Y205A mutation abolishing the binding to LDL is that the Y205A mutation causes a conformational transition. The Y205 residue provides an important interaction between helix 101–111 to the rest of the protein via a cation-pi interaction with K109. Replacement of Y205 with alanine, disrupts the interaction with K109, as a result helix 101–111 may move relative to the beta-hairpin 186–205. Next to helix 101–111 is helix 115–125, which is one of the i-face helices. Conformational changes, even subtle, of this important helix would explain how the Y205A mutation abolishes LDL-binding. This hypothesis and a full structural explanation could be tested by binding studies performed with a K109 mutant and by solution of a crystal structure of the Y205A mutant. In the absence of experimental data, although unlikely, it cannot be ruled out that Y205 mediates association with LDL; instead of interacting with the phospholipid monolayer, it could be interacting with apoB100. 

### 5.3. Role of Glycosylation in the Interaction of Plasma PAF-AH with Lipoproteins

As mentioned earlier, N-linked glycosylation of plasma PAF-AH was shown to hinder its binding to HDL in humans [25]. The predicted sites of glycosylation in human plasma PAF-AH are, N423 and N433. It is interesting that these residues are not conserved in other species such as mouse (Figure 3) in which the enzyme associates exclusively with HDL. It should be noted however that glycosylation of plasma PAF-AH in species other than humans has not been demonstrated experimentally. Since the structure of a non-glycosylated form of the enzyme was solved, it is difficult to speculate how glycosylation might be affecting binding to HDL. In the crystal structure of PAF-AH, N423 lies far from the i-face region but is close to the acidic patch region, which is described below. The structural position of N433 is not known, since the structure of a shorter PAF-AH construct lacking N433 was solved [7]. The current explanation for the observed effect of glycosylation in PAF-AH on lipoprotein association is that it hinders association with HDL particles. An alternate explanation is that glycosylation actually promotes binding to LDL particles. In this scenario, the absence of glycosylation would take away the ability of LDL to attract PAF-AH preferentially over HDL, and as a result the enzyme then associates equally with both the lipoproteins.

### 5.4. Role of Charged Amino Acids in the Interaction with Lipoproteins

In addition to the phospholipid monolayer of the lipoprotein particles, the protein component of lipoprotein particles are also involved in the interaction with plasma PAF-AH. As already mentioned, the C-terminal region of apolipoprotein present on LDL – apoB100 has been shown to be involved in the association of plasma PAF-AH with LDL. The major apolipoprotein present on HDL is apolipoprotein A-I (apoA-I); some HDL particles also have apolipoprotein A-II (apoA-II) in addition to apoA-I [47]. A still smaller percentage of HDL particles have other additional proteins such as, apoCs, apoD, apoE, apoM and apoA-IV. It is possible that the type of interaction between plasma PAF-AH amino acids and the amino acids of the apolipoproteins are electrostatic in nature. 

**Figure 4:**
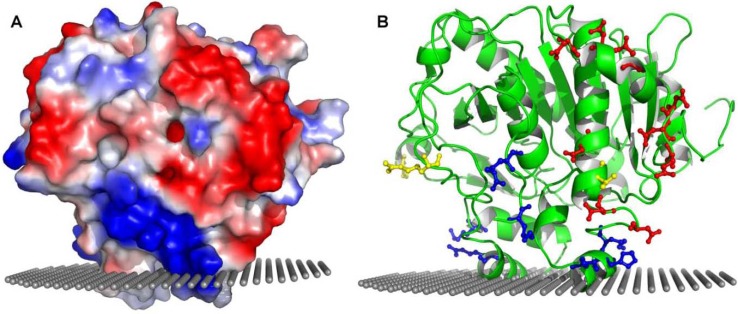
Acidic and basic patches in PAF-AH. Panel A depicts the surface electrostatic potential view of PAF-AH showing the acidic and one of the basic patches. Panel B on the right shows a ribbon diagram of PAF-AH in roughly the same orientation as in panel A. Amino acids comprising the acidic and basic patches are highlighted in red and blue, respectively. Three naturally occurring non-synonymous polymorphisms (R92H, I198T, A379V) are highlighted in yellow, with the V379 residue shown at the base of the acidic patch.

The region in PAF-AH that is involved in its interaction with apoB100 has not yet been identified. The crystal structure of PAF-AH reveals a stretch of negatively charged amino acids, an acidic patch, which could potentially be involved in the interaction with apoB100 (Table 1 and Figure 4). 

ApoB100 is known to contain clusters of positively charged residues that mediate the electrostatic interaction of LDL with the negatively charged sulfate groups of glycosaminoglycans (GAGs) [48,49,50]. These clusters of positively charged amino acids may also mediate an electrostatic interaction with the acidic patch of PAF-AH. The increased affinity of PAF-AH for small dense LDL, LDL(-) and Lp(a) is proposed to be mediated by the altered conformation of apoB100. It is possible that a change in the conformation of apoB100 in these lipoproteins exposes additional regions of positively charged residues which would increase the affinity towards the negatively charged acidic patch of PAF-AH. In addition, it is possible that the interaction between apoB100 and plasma PAF-AH also plays a role in the unequal distribution of PAF-AH between LDL and HDL. Since apoB100 is present only on LDL and not HDL, this interaction could potentially tip the balance in favor of LDL particles.

Several plasma PAF-AH polymorphisms have been identified in different populations [51,52]. Of these, only V279F and Q281R have been found to result in loss of enzyme activity and have been shown to be linked to human diseases [53,54,55]. Three other mutations that result in active enzyme and have been studied to determine their correlation with the incidence of several human diseases are, R92H, I198T, A379V (reviewed in [56]). Each of these three polymorphic sites are solvent exposed and are far from the active site. It is possible that the phenotypic differences that are exhibited in individuals carrying these polymorphic alleles are due to the differences in the interactions with lipoproteins. Notably, amino acid 379 is located close to the acidic patch region (Figure 4, panel B). The presence of alanine vs. valine at this position has been suggested to be significant, with correlation to cardiovascular diseases [57,58,59]. It is possible that the observed correlation is due to the modulation of the distribution of the enzyme between LDL and HDL particles.

In addition to the cluster of negatively charged amino acids, two clusters of basic patches are also apparent from the surface electrostatic map of PAF-AH (Table 1, Figure 4). The basic patch amino acids could either be interacting with the protein component of LDL or HDL particles, or they could be interacting with the phosphoryl groups of phospholipids in both the lipoproteins. Basic amino acids are known to mediate the membrane association of many peripheral membrane proteins, either through non-specific or specific electrostatic interaction with the negatively charged phosphoryl groups of phospholipids [60]. They are usually in the vicinity of the hydrophobic residues that insert into the interface. H367 and K370, as mentioned earlier, form part of a HMLK region that was shown to mediate the association of PAF-AH with HDL. 

As in the case of other peripheral membrane proteins, these electrostatic interactions may be the first step to bring the enzyme close to the lipoproteins. Once they are in proximity, a more stable enzyme-lipoprotein complex is formed aided by the membrane insertion of hydrophobic residues. Long-range electrostatic attraction is believed to increase the probability of a protein-membrane interaction and helps orient the protein. While the basic and hydrophobic residues might be involved in the interaction with the lipid component of both lipoprotein molecules, a likely scenario is that the acidic patch region interacts with solely the protein component of LDL particles. Amino acids of both the acidic patch and the basic patch are fairly well conserved when compared across mammalian species, but as expected are not conserved in the cytoplasmic PAF-AH II (Figure 3). The additional features of glycosylation and electrostatic interaction mediated by the cluster of negatively charged amino acids in PAF-AH may provide specificity for one type of lipoprotein over another. It is possible that in the absence of these features, PAF-AH would not distinguish between LDL and HDL. 

### 5.5. Number of Plasma PAF-AH Molecule Per Lipoprotein Molecule

The limiting factor that dictates the number of PAF-AH molecules that can bind to each molecule of LDL is likely to be the surface pressure in the phospholipid monolayer. The surface of lipoprotein particles is more rigid than a membrane bilayer due to the presence of lipoproteins and only a monolayer of phospholipids instead of bilayer. Due to a steadily increasing surface pressure, the insertion of each successive PAF-AH molecule is predicted to be more and more difficult. However, the physiological concentration of PAF-AH is much lower than this limit, as indicated by the molar ratio of PAF-AH to apoB100. The molar ratio of PAF-AH to apoB100 has been reported to vary from 1:100 to 1:10,000. Even in the LDL subfractions that are enriched in PAF-AH, such as small dense LDL(-), only 1% of the lipoproteins have a PAF-AH molecule bound, and as a result most particles do not contain PAF-AH [34]. The equilibrium of PAF-AH with HDL and LDL particles in a physiologically relevant environment would be further complicated by a complex equilibrium involving many other membrane associated proteins competing with PAF-AH for a chance to bind the lipoproteins.

## 6. Conclusions

In this review we have summarized the current understanding of the interaction between PAF-AH and lipoproteins. Although regions of PAF-AH that mediate its association with LDL and HDL particles have been identified, the specific role played by the amino acids had not been clearly understood. Using the structure as a guide, we have presented a hypothesis on how each of the amino acids identified earlier interacts with lipoproteins. We have proposed that hydrophobic and aromatic residues in PAF-AH, which were previously identified as distinct LDL and HDL binding regions, together form the i-face. Amino acids comprising the i-face insert into the phospholipid monolayer and anchor the enzyme on the surface of lipoproteins. Additional factors may influence the preference of PAF-AH for LDL over HDL. Electrostatic interaction between a pronounced acidic patch of PAF-AH and positively charged amino acids on the surface of apoB100 is potentially one such factor. The increased affinity of PAF-AH for LDL subclasses, small dense LDL, LDL(-) and Lp(a), can also be explained by this electrostatic interaction. Additionally, a group of basic amino acids near the i-face may mediate an electrostatic interaction with the phospholipid head groups or with the protein component of the lipoproteins. It is likely that distinct amino acids in PAF-AH mediate interaction with both the lipid and protein components of lipoproteins. Although many questions about the interaction of PAF-AH with lipoproteins still remain unanswered, with the crystal structure of PAF-AH now available we are beginning to better understand the association of PAF-AH with lipoproteins.
